# Continuity of care for patients recovering from Covid-19 under the angle of clinical management principles

**DOI:** 10.1590/1980-220X-REEUSP-2023-0123en

**Published:** 2023-11-20

**Authors:** Vanessa Alves Mendes, Maria Fernanda Baeta Neves Alonso da Costa, Anna Flávia da Silva Almeida Martins, Karina Nonato Mocheuti, Gímerson Erick Ferreira, Mara Regina Rosa Ribeiro

**Affiliations:** 1 Universidade Federal de Mato Grosso, Faculdade de Enfermagem, Cuiabá, MT, Brasil.; 2 Universidade Federal de Santa Catarina, Programa de Pós-Graduação em Enfermagem, Florianópolis, SC, Brasil.

**Keywords:** Continuity of Patient Care, Patient Discharge, Covid-19, Nursing Care, Patient-Centered Care, Continuidad de la Atención al Paciente, Alta del Paciente, Covid-19, Atención de Enfermería, Atención Dirigida al Paciente, Continuidade da Assistência ao Paciente, Alta do Paciente, Covid-19, Cuidados de Enfermagem, Assistência Centrada no Paciente

## Abstract

**Objective:**

To analyze the strategies used by nurses at a university hospital to ensure continuity of care at hospital discharge for patients recovered from Covid-19, under the angle of the principles of clinical management.

**Method:**

A descriptive study with a qualitative approach, carried out with seven nurses on duty in the medical and gynecology/obstetrics clinics of a university hospital in the Midwest region of the country. The data was processed using IRaMuTeQ software and analyzed using Content Analysis.

**Results:**

The data resulted in five classes by the Descending Hierarchical Classification (DHC), which made up two categories: “Practices developed by nurses for continuity of care in the hospital environment” and “Continuity of care during discharge to the home”. The strategies used by the nurses were: daily care systematized in the nursing process and guidance both for preparation and for the day of discharge.

**Conclusion:**

The absence of an institutional protocol for safe discharge, as well as the position of nurse coordinator to manage the discharge of patients with Covid-19, can compromise the continuity of care for these patients.

## RESUMO

**Objetivo:**

Analisar as estratégias utilizadas por enfermeiros de um hospital universitário para garantir continuidade do cuidado na alta hospitalar de pacientes recuperados da Covid-19, à luz dos princípios da gestão da clínica.

**Método:**

Estudo descritivo de abordagem qualitativa, realizado com sete enfermeiros plantonistas das clínicas médicas e ginecologia/obstetrícia de um hospital universitário da região centro-oeste do país. Os dados foram processados pelo software IRaMuTeQ e analisados pelo método de Análise de Conteúdo.

**Resultados:**

Os dados resultaram em cinco classes pela Classificação Hierárquica Descendente (CHD), que compôs duas categorias: “Práticas desenvolvidas pelos enfermeiros para a continuidade do cuidado no ambiente hospitalar” e “Continuidade do cuidado durante a alta hospitalar para o domicílio”. As estratégias utilizadas pelos enfermeiros foram: cuidados diários sistematizados no processo de enfermagem e orientações tanto para o preparo quanto para o dia de alta.

**Conclusão:**

A ausência de protocolo institucional para alta segura, bem como o cargo de enfermeira coordenadora para gerenciar as altas dos pacientes com Covid-19 podem comprometer a continuidade do cuidado a esses pacientes.

## RESUMEN

**Objetivo:**

Analizar las estrategias utilizadas por el personal de enfermería de un hospital universitario para asegurar la continuidad asistencial al alta hospitalaria de pacientes recuperadas de Covid-19, a la luz de los principios de gestión clínica.

**Método:**

Estudio descriptivo con abordaje cualitativo, realizado con siete enfermeras de guardia en las consultas de medicina y ginecología/obstetricia de un hospital universitario de la región centro-oeste del país. Los datos se procesaron con el programa IRaMuTeQ y se analizaron mediante Análisis de Contenido.

**Resultados:**

Los datos resultaron en cinco clases por la Clasificación Jerárquica Descendente (CJD), que constituyeron dos categorías: “Prácticas desarrolladas por las enfermeras para la continuidad de los cuidados en el ambiente hospitalario” y “Continuidad de los cuidados durante el alta al domicilio”. Las estrategias utilizadas por las enfermeras fueron: cuidados diarios sistematizados en el proceso de enfermería y orientación tanto para la preparación como para el día del alta.

**Conclusión:**

La ausencia de un protocolo institucional para el alta segura, así como del cargo de enfermera coordinadora para gestionar el alta de pacientes con covid-19, puede comprometer la continuidad de cuidados de estos pacientes.

## INTRODUCTION

To think about continuity of care is a challenging task whenever patients change health care settings, as they run the risk of getting lost in the care arrangement due to a lack of knowledge about the services they need^(1)^. This moment of transition of care, understood as coordinated and effective actions to ensure continuity of care when transferring the patient between different health services at hospital discharge^(2,3)^, is a period of vulnerability, and it is important for health professionals to plan for hospital discharge, post-discharge follow-up and guidelines for care at home, in order to improve the quality of care outcomes and influence patients’ quality of life, preventing readmissions and, consequently, reducing unnecessary hospital costs^(1–3)^.

The role of nurses during hospital discharge is fundamental to guaranteeing success in the transition process to return home, through articulation and communication between health services^(2)^, experiences on the international scene are successful^(1,4)^. However, the following are still challenges encountered in Brazilian health services: the absence of a hospital discharge planning protocol^(5)^, lack of referral and counter-referral to health services^(6)^ and nurses’ professional practice limited to guidance which is sometimes given quickly and within a short period of time, due to the workload with numerous activities to be completed^(7)^.

Associated with these challenges, at the beginning of 2020, health institutions across the country experienced a major public health challenge, the pandemic caused by the severe acute respiratory syndrome coronavirus 2 (SARS-COv-2), responsible for an infection with symptoms ranging from mild to severe, with a high mortality rate, a high bed occupancy rate, and an exponential increase in the demand for material and human resources to care for patients affected by the infection^(8,9,10)^.

In this pandemic scenario, investments have been made in health services to increase the number of beds^(11)^, purchase supplies, hire new professionals, develop training actions for the health team^(12)^, and reorganize care processes^(13)^. Due to the severity of the disease and the risk of patients suffering sequelae, systematized and multidisciplinary follow-up after hospital discharge has become more necessary in order to guarantee better health recovery^(14)^.

Strategies such as safe discharge, bed management, clinical management, case discussions between the multidisciplinary team, continuing health education and a nurse responsible for coordinating discharges are some of the strategies highlighted in the literature to ensure continuity of care inside and outside the hospital environment^(15)^. In the meantime, this study is articulated with the theoretical approach of Clinical Management^(16)^, which consists of a set of micro-management technologies based on scientific evidence, aimed at ensuring quality, safe, shared, person-centered health care and guided by better performance standards, which is structured on principles that articulate with each other in the dimensions of management, care and education^(17)^. As such, it is a model of care with the strategic potential to transversalize the different points of the Healthcare Network, in order to provide comprehensive, effective and continuous care.

Although the disease has been widely publicized in scientific circles, little is known about how patients who have recovered from Covid-19 have been followed up after being discharged from hospital. Given the unprecedented nature of the disease, these patients need to be followed up to understand how their health and quality of life were restored. Nurses are considered to be the most qualified professionals to ensure continuity of care^(5)^. Therefore, the question of this study involves investigating which strategies nurses use to ensure continuity of care for patients with Covid-19 on discharge from hospital to home.

This study is part of the national multicenter project entitled “Evaluation of nursing care for patients with Covid-19 in Brazilian university hospitals”. In view of the above, the aim is to analyze the strategies used by nurses at a university hospital to ensure continuity of care at hospital discharge for patients recovered from Covid-19, in the light of the principles of clinical management.

## METHOD

### Study Design

This is a descriptive-exploratory study of a qualitative nature, structured based on the Consolidated Criteria for Reporting Qualitative Research (COREQ) Guide^(18)^.

### Population, Location and Selection Criteria

The participants were nurses on shifts in the clinical inpatient units, namely: medical, gynecological and obstetric. Of the eight nurses who made up the clinical team, seven responded to the invitation to participate, four of whom worked in the medical clinic and three in gynecology and obstetrics. The inclusion of nurses was based on the following criteria: having experience in caring for patients with Covid-19, working on day shift, as this is the period that best prepares and monitors hospital discharges. Professionals on vacation or on leave of any kind during the data production period were excluded.

The setting for the study was a university hospital in the Midwest region of Brazil, considered a reference in the care of patients affected by Covid-19, under the management responsibility of the Brazilian Hospital Services Company EBSERH. The inpatient units selected are justified because they have been a reference in the state for the care of patients with suspected or confirmed diagnosis of Covid-19, as well as receiving those patients recovered from the severe state of the disease coming from the intensive care unit (ICU).

### Data Collection

Data was collected from November 2021 to February 2022, face-to-face, in the professionals’ work environment, using a semi-structured interview script consisting of professional characterization questions and questions related to the care provided by nurses to patients with Covid-19, during hospital admission, hospitalization, preparation for discharge, regarding the guidance provided to the patient, family or caregivers; contact with Primary Health Care, in addition to complementary questions if there was a discharge plan at the institution. The interviews were recorded on digital audio media by two trained researchers and lasted an average of 30 minutes. The empirical material obtained from the interviews was transcribed in full using WORD software, organized into individual files and returned to the participants for approval of the transcribed content.

### Data Analysis and Processing

The data was organized and managed using the IRaMuTeQ software (Interface de R pour les Analyses Multidimensionnelles de Textes et de Questionnaires). The Reinet method was adopted to help construct the classes, with 85.9% of the textual corpus being used, in a time of one minute and 12 seconds. For data analysis, we used the thematic content analysis proposed by Bardin^(19)^, going through three phases: (1) pre-analysis; (2) exploration of the material and (3) treatment of the results, inference and interpretation.

### Ethical Aspects

This study is part of a multicenter project that aimed to evaluate nursing care for patients with Covid-19 in university hospitals in all regions of the country, approved in a public call (nº 005/2020 – nº07/2020) for research to tackle Covid-19, its consequences and other severe acute respiratory syndromes (Process nº: 402392/2020-5). All the research complied with the recommendations of Resolution 466, of December 12, 2012, of the National Health Council, which approves guidelines and regulatory standards for research involving human beings. The matrix project was approved by the Human Research Ethics Committee (CEP) of the Federal University of Santa Catarina under protocol no. 4023392/2020-5, in addition to this, there was approval by the local CEP in December 2020, under opinion no. 4.466.821, and the Free and Informed Consent Form - TCLE was previously sent for prior reading by the participants, informed about the objectives, risks and benefits of their participation, to agree to voluntarily participate in the interviews and their recording. The confidentiality and anonymity of the participants was guaranteed through the code composed of the letter E, followed by an ordinal number (Example: E1).

## RESULTS

The interviewees’ ages ranged from 27.8 to 39.1 years. With regard to gender, only one was male, the length of time working in the hospital unit ranged from four months to seven years, all professionals are part of the permanent staff hired by public tender, with experience of working with Covid-19 since March 2020. As for the level of training, the highest degree was a master’s degree for two interviewees, the others were specialists and graduates.

**Figure 1:**
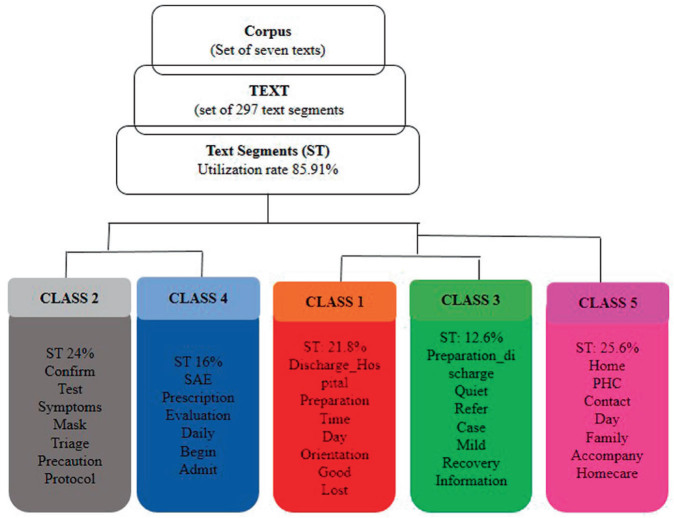
Dendrogram of the textual corpus of the interviews.

Source: Research data, 2022.

The processing of the textual corpus generated five classes according to the dendrogram (Figure 1). We opted for the Descending Hierarchical Classification (DHC) which provided two subdivisions based on the segments: at the top of the dendrogram are classes 2 (ST = 24%) and 4 (ST = 16%), while classes 1 (ST = 21.6%) and 3 (ST = 12.8%) have converging vocabularies, which are related to class 5 (ST = 25.6%), in the second division.

Two categories emerged from the analysis, the first being “Practices developed by nurses for continuity of care in the hospital environment”, which revealed the technical care provided by the professional, the systematization of this care through the nursing process, as well as the professional’s communication with the nursing team and other professional categories (Chart 1).

**Chart 1:** Categories and subcategories generated from the interviewees’ statements – Midwest region, Brazil, 2022.

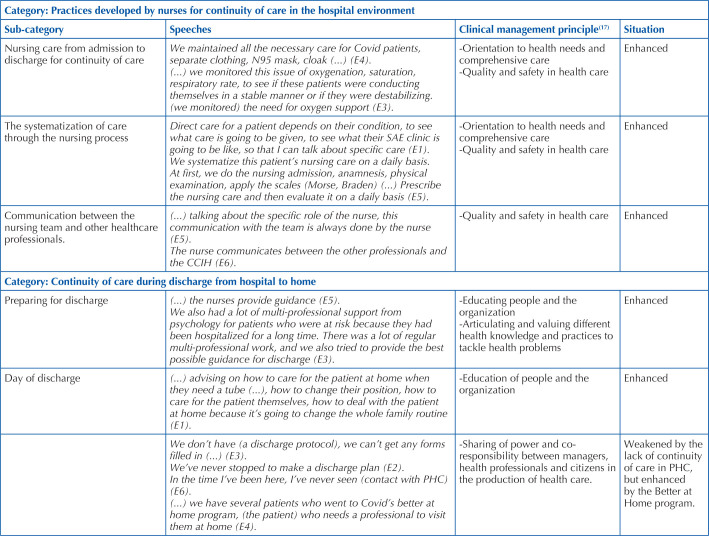

Source: Research data, 2022.

The second category was “Continuity of care during discharge from hospital to home”, in which practitioners discussed aspects of preparation for discharge based briefly on guidance, with preparation time differing from case to case, as in some situations nurses are made aware of the discharge in advance by the medical team. On the other hand, there are cases in which the nursing team is notified after the patient has been discharged, challenging the nurses to give the patient insufficient guidance before the patient leaves.

On the day of discharge, the role highlighted by the interviewees was based on guidance for the patient and family, depending on the clinical condition at the time of leaving hospital, as there were cases of patients who went home with sequelae from Covid-19, requiring more complex care and support from home care. For those patients without sequelae, the advice was limited to precautionary measures to prevent re-infection or transmission to family members. The work of the multidisciplinary team in this process should be highlighted, and the importance of working together with other professionals for successful preparation was commented on. Furthermore, the lack of an institutional protocol for hospital discharge was eloquent.

## DISCUSSION

The strategies employed by nurses to guarantee continuity of care for patients recovering from Covid-19 during discharge from hospital to home were: systematization of nursing care through the nursing process; guidance on care for patients and family members during discharge from hospital, as well as home care after leaving the hospital environment. The lack of a discharge protocol was highlighted as a challenge to promoting continuous care. The principles of the clinical management model^(17)^ present important elements to promote continuity of care, based on the triad of management, education and assistance, in the search for comprehensive, safe, quality healthcare aimed at meeting the needs of the user.

In this study, discharges in the context analyzed are managed by the care nurse, who sometimes has to give discharge instructions in the midst of the day-to-day tasks on duty. Discharge planning is an educational and preventive process that should begin within 24 hours of the patient’s admission^(5)^ and the participation of nurses is essential to ensure continuity of care^(1)^.

The nurses in the study demonstrate that they understand the importance of daily guidance as a way of preparing the patient for discharge. However, as in other Brazilian contexts^(7)^, it is not a practice to carry out discharge planning, associating this with inefficient communication from the medical team, the lack of institutional protocols, as well as the numerous care duties on duty as factors that weaken the follow-up of this patient until discharge.

Having a professional nurse responsible for managing hospital discharges, known as liaison nurses, is an efficient strategy for reducing the problems arising from the lack of communication between health care service points and promoting continuity of care^(3,20)^. The activities carried out by these professionals focus on the patient’s needs, through guidance on self-care, provision of the necessary resources for care at home and coordination with out-of-hospital services, especially PHC^(21)^. Therefore, the absence of a liaison nurse in the context studied may be a factor capable of fragmenting care, causing negative effects on the quality of care, as well as favoring hospital readmissions^(22)^.

The lack of integration between the hospital and primary care is a negative aspect in the studied context, diverging from the ideal model recommended in the literature^(23)^, but it cannot be said that continuity of care does not exist. The lack of counter-referrals in Brazilian health services is still commonplace in health care settings^(20)^. Although the Covid-19 pandemic requires highly complex care in the vast majority of cases, the authors point out that PHC is an important pillar for dealing with emergency situations^(24)^.

Professionals working in this care setting must be able to provide comprehensive primary care, disseminating resources for decision-making when possible complications and/or new cases are detected, preventing new hospital breakdowns^(25)^, as well as ensuring continuity of care according to the user’s needs after hospital discharge. However, when it comes to continuity of care, PHC is usually seen as responsible for supplying health materials and devices, rarely as an aspect of care in counter-referrals, and is more likely to seek support from home-based services^(23)^.

Home care, as a type of health care integrated into the health care network, especially PHC, is characterized as a set of promotion, prevention, treatment and rehabilitation actions, in order to guarantee continuity of care^(26)^. Home care is an attribute of PHC^(27)^. The most stable patients, who require little care, can be followed up by basic health units. On the other hand, more complex cases with a higher frequency of care, health resources and continuous monitoring should be monitored by a multi-professional home care team (EMAD in the Portuguese acronym) and multi-professional support team (EMAP in the Portuguese acronym) included in the Better at Home Program, based on shared care between family members and/or responsible caregivers^(28)^.

The nurses interviewed revealed the potential of the Better at Home Program for continuity of care. Patients who require home care are discharged with a visit from the EMAD team for treatment and rehabilitation at home. The power of home care during the pandemic should be highlighted, given the risk of the health system collapsing due to the overload and overcrowding of beds in hospitals working at maximum capacity^(27)^.

Therefore, home care, by virtue of carrying out responsible discharge and assessing the patient’s clinical condition for continuity of care by the multidisciplinary team, proves to be effective in interrupting the transmission of the virus, by reducing the circulation of people outside the home; identifying, isolating and caring for new cases early; as well as making hospital beds available, reinforcing that this type of care is capable of solving the health needs of individuals with effectiveness of services and defragmentation^(27,29)^.

The analysis of the narratives points to a challenge in nursing practice in relation to communication with the medical team at the time of discharge, which can hinder continuity of care, as the nurse is not informed in advance of discharge, leaving little time to guide the patient before they leave. Communication and collaboration between the professionals who care for the patient are highlighted as challenges to meeting the relational dimension for continuity of care in nursing practice^(30)^.

Efficient communication and collaborative relationships between professionals who care for patients are fundamental elements for continuity of care^(22)^. The findings of this study show that nurses enhance this practice by being the professional who communicates with the nursing team and other professionals in the hospital context. It is believed that the nurse’s professional practice based on leadership and trust is a way of making other professionals understand the nurse’s competencies in the continuity of care, respecting the space and functions of the other professionals involved in the care process according to the individual needs of each patient^(22)^.

In addition to this communication between the nurse-patient, family member and health professionals from the multidisciplinary team, there are other essential elements to ensure continuity of care, namely: ensuring access to care based on the needs of the individual, establishing flexibility in the organization and structural possibilities for the nurse to plan and create space for visits to patients; making professionals responsible as producers of care for the patient: as well as establishing a relationship of trust between all the subjects involved in the care process^(30)^.

It was possible to identify the principles of the clinical management approach. Of the seven principles described by^(16)^, five were notably practiced in the hospital context studied, favoring health care geared to the health needs of the individual, articulated between managers, professionals and users for comprehensive, safe and quality care. The most prominent principle was “Orientation to health needs and comprehensive care”. The presence of a companion for dependent patients was the care practice with the greatest health needs at the time.

In relation to “Sharing power and co-responsibility between managers, health professionals and citizens in the production of health care”, which refers to the co-responsibility and articulation of services-professionals at the different levels of care, it was a principle that shows weaknesses when observed the lack of communication between the hospital and PHC. However, for the time being it has been strengthened by the actions of the Better at Home Program, home care, in which managers and health professionals build common objectives, with criticality and commitment, in favor of comprehensive care and in accordance with the needs of the person being cared for.

The limitation of this study is the absence of a nurse coordinator of discharge at the study site, which makes it difficult to investigate the continuity of care from the hospital setting to the home. Another limitation refers to the local setting of a teaching hospital, so generalizations are not possible. However, in order to explore the questions and deepen the experiences, the nurses invited were all those who worked with Covid-19 patients in the inpatient clinics at the institution at study.

## CONCLUSION

The systematization of nursing care based on the nursing process, as well as daily guidelines capable of supporting preparation for discharge and leaving the hospital for home, were the strategies reported by the interviewees as enhancing the continuity of care. The lack of an institutional protocol for safe discharge, of a nurse to manage the discharge of patients with Covid-19 and of counter-referral with the basic health service, can compromise the continuity of care for these patients. It is extremely important that health services are articulated, so that teams are able to communicate between the different levels of services, so that the patient is discharged from hospital and health care is ensured according to their needs in primary health care or home care.

As a contribution to nursing, it is believed that this study provides information on the potential and weaknesses of nurses’ actions to promote continuity of care at a complex time of change between health care settings, which can make comprehensive care unfeasible due to the lack of coordinated, person-centered actions. Considering the fragility in terms of instrumentalizing hospital discharge observed in this study, it is recommended that further studies be carried out with a view to building tools for hospital discharge, as well as follow-up studies of post-Covid-19 patients after leaving hospital, to identify sequelae or contributors to recovery and maintaining the individual’s quality of life.

## References

[1] Costa MFBNA, Perez EIB, Ciosak SI (2021). Practices of hospital nurses for continuity of care in primary care: an exploratory study. Texto Contexto Enferm.

[2] Weber LAF, Lima MADDS, Acosta AM, Marques GQ (2017). Cogitare Enfermagem.

[3] Bernardino E, Sousa SMD, Nascimento JDD, Lacerda MR, Torres DG, Gonçalves LS (2021). Cuidados de transição: análise do conceito na gestão da alta hospitalar. Esc Anna Nery.

[4] Costa MFBNAD, Andrade SRD, Soares CF, Pérez EIB, Tomás SC, Bernardino E (2019). A continuidade do cuidado de enfermagem hospitalar para a Atenção Primaria à Saúde na Espanha. Rev Esc Enferm USP.

[5] Nunes ECDA, Menezes Fo NA (2016). Sistematização da alta de enfermagem: uma análise fundamentada em Roy. Cogitare Enfermagem.

[6] Brondani JE, Leal FZ, Potter C, Silva RM, Noal HC, Silveira Perrando M (2016). Desafios da referência e contrarreferência na atenção em saúde na perspectiva dos trabalhadores. Cogitare Enfermagem.

[7] Acosta AM, Câmara CE, Weber LAF, Fontenele RM (2018). Atividades do enfermeiro na transição do cuidado: realidades e desafios. Revista de Enfermagem UFPE.

[8] Ciotti M, Ciccozzi M, Terrinoni A, Jiang WC, Wang CB, Bernardini S (2020). The Covid-19 pandemic. Crit Rev Clin Lab Sci.

[9] Sardinha DM, Lima KVB, Ueno TMRL, Rodrigues YC, Garcez JCD, Santos ALS (2021). Occurrence of cardiovascular complications associated with SARS-CoV-2 infection: a systematic review. J Pharm Res Int.

[10] Silva GA, Jardim BC, Santos CVB (2020). Excesso de mortalidade no Brasil em tempos de Covid-19. Cien Saude Colet.

[11] Santos JLGD, Menegon FHA, Andrade GB, Freitas EO, Camponogara S, Balsanelli AP (2021). Changes implemented in the work environment of nurses in the Covid-19 pandemic. Rev Bras Enferm.

[12] Santos JLGD, Lanzoni GMDM, Costa MFBNAD, Debetio JO, Sousa LPD, Santos LSD (2020). Coo os hospitais universitários estão enfrentando a pandemia de COVID-19 no Brasil?. Acta Paul Enferm.

[13] Medeiros EAS (2020). Challenges in the fight against the Covid-19 pandemic in University hospitals. Rev Paul Pediatr.

[14] Graça NP, Viscont NRGR, Santos MIV, Capone D, Cardoso AP, Mello FCQ (2020). COVID-19: seguimento após a alta hospitalar. Pulmão RJ.

[15] Belga SMMF, Jorge AO, Silva KL (2022). Continuidade do cuidado a partir do hospital: interdisciplinaridade e dispositivos para integralidade na rede de atenção à saúde. Saúde Debate.

[16] Mendes EV (2011). As Redes de Atenção.

[17] Padilha RQ, Gomes R, Lima VV, Soeiro E, Oliveira JM, Schiesari LMC (2018). Princípios para a gestão da clínica: conectando gestão, atenção à saúde e educação na saúde. Cien Saude Colet.

[18] Souza VRDS, Marziale MHP, Silva GTR, Nascimento PL (2021). Tradução e validação para a língua portuguesa e avaliação do guia COREQ. Acta Paul Enferm.

[19] Bardin L (2016). Análise de conteúdo.

[20] Ribas EDN, Bernardino E, Larocca LM, Poli Neto P, Aued GK, Silva CPCD (2018). Nurse liaison: a strategy for counter-referral. Rev Bras Enferm.

[21] David HMSL, Riera JRM, Mallebrera AH, Costa MFLD (2020). A enfermeira gestora de casos na Espanha: enfrentando o desafio da cronicidade por meio de uma prática integral. Cien Saude Colet.

[22] García-Vivar C, Soto-Ruiz N, Escalada-Hernández P, Ferraz-Torres M, Orzanco-Garralda MR, Martín-Rodríguez LS (2022). Continuity of care challenges for professional nursing practice. Aquichan.

[23] Utzumi FC, Bernardino E, Lacerda MR, Santos JLG, Peres AM, Andrade SR (2020). Acess versus care continuity in health network services: experiencing possibilities and contradictions. Texto Contexto Enferm.

[24] Dunlop C, Howe A, Li D, Allen LN (2020). The coronavirus outbreak: the central role of primary care in emergency preparedness and response. BJGP Open.

[25] Sarti TD, Lazarini WS, Fontenelle LF, Almeida APSC (2020). Qual o papel da Atenção Primária à Saúde diante da pandemia provocada pela Covid-19?. Epidemiol Serv Saude.

[26] Ministério da Saúde (2016). Portaria No 825..

[27] Savassi LCM, Reis GVL, Dias MB, Vilela LO, Ribeiro MTAM, Zachi MLR (2020). Recomendações para a Atenção Domiciliar em período de pandemia por Covid-19. Rev Bras Med Fam Comunidade.

[28] Ministério da Saúde (2020). Nota técnica N^o^ 9/2020-CGAHD/DAHU/SAES/MS.

[29] Castro EAB, Leone DRR, Santos CM (2018). Organização da atenção domiciliar com o Programa Melhor em Casa. Rev Gaúcha Enferm.

[30] Östman M, Bäck-Pettersson S, Sundler AJ, Sandvik AH (2021). Nurses’ experiences of continuity of care for patients with heart failure: A thematic analysis. J Clin Nurs.

